# A 2D graphical representation of the sequences of DNA based on triplets and its application

**DOI:** 10.1186/1687-4153-2014-1

**Published:** 2014-01-02

**Authors:** Sai Zou, Lei Wang, Junfeng Wang

**Affiliations:** 1 School of Software Engineering, Chongqing College of Electronic Engineering, Chongqing 401331, People's Republic of China

**Keywords:** Graphical representation, Similarities/dissimilarities analysis, Triplet, DNA sequence

## Abstract

In this paper, we first present a new concept of ‘weight’ for 64 triplets and define a different weight for each kind of triplet. Then, we give a novel 2D graphical representation for DNA sequences, which can transform a DNA sequence into a plot set to facilitate quantitative comparisons of DNA sequences. Thereafter, associating with a newly designed measure of similarity, we introduce a novel approach to make similarities/dissimilarities analysis of DNA sequences. Finally, the applications in similarities/dissimilarities analysis of the complete coding sequences of β-globin genes of 11 species illustrate the utilities of our newly proposed method.

## 1. Introduction

In the recent years, an exponential growth of sequence data in DNA databases has been observed by biologists; the importance of understanding genetic sequences coupled with the difficulty of working with such immense volumes of DNA sequence data underscores the urgent need for supportive visual tools. Recently, graphical representation is well regarded which can offer visual inspection of data and provide a simple way to facilitate the similarity analysis and comparison of DNA sequences [1-5]. Because of its convenience and excellent maneuverability, currently, all kinds of methods based on graphical representation have been extensively applied in relevant realms of bioinformatics.

Until now, there are many different graphical representation methods having been proposed to numerically characterize DNA sequences on the basis of different multiple-dimension spaces. For example, Liao et al. [6-9], Randic et al. [10-13], Guo et al. [14,15], Qi et al. [16], Dai et al. [17,18], and Dorota et al. [19] proposed different 2D graphical representation methods of DNA sequences, respectively. Liao et al. [20-23], Randic et al. [24,25], Qi et al. [26], Yu et al. [27], and Aram et al. [28] proposed different 3D graphical representation methods of DNA sequences, respectively. Liao et al. [29], Tang et al. [30], and Chi et al. [31] proposed different 4D graphical representation methods of DNA sequences, respectively. In addition, Liao et al. [32] also proposed a kind of 5D representation method of DNA sequences and so on.

In these approaches mentioned above, most of them adopt the leading eigenvalues of some matrices, such as *L*/*L* matrices, *M*/*M* matrices, *E* matrices, covariance matrices, and *D*/*D* matrices, to weigh the similarities/dissimilarities among the complete coding sequences of β-globin genes of different species. Because the matrix computation is needed to obtain the leading eigenvalues, these methods are usually computationally expensive for long DNA sequences. Furthermore, in some of these approaches, their results of similarities/dissimilarities analysis are not quite reasonable, and there are some results that do not accord with the fact [7,9].

To degrade the computational complexity and obtain more reasonable results of similarities/dissimilarities analysis of DNA sequences, in this article, we propose a new 2D graphical representation of DNA sequences based on triplets, in which, we present a new concept of ‘weight’ for 64 triplets and a new concept of ‘weight deviation’ to weigh the similarities/dissimilarities among the complete coding sequences of β-globin genes of different species. Compared with some existing graphical representations of the DNA sequences, our new scheme has the following advantages: (1) no matrix computation is needed, and (2) it can characterize the graphical representations for DNA sequences exactly and obtain reasonable results of similarities/dissimilarities analysis of DNA sequences.

## 2. Proposed 2D graphical representation of DNA sequence

Codon is a specific sequence of three adjacent nucleotides on the mRNA that specifies the genetic code information for synthesizing a particular amino acid. As illustrated in Table 1, there are total 20 amino acids and 64 codons in the natural world, and each of these codons has a specific meaning in protein synthesis: 64 codons represent amino acids and the other 3 codons cause the termination of protein synthesis.

**Table 1:** Relationship between 20 different kinds of most common amino acids and 64 different kinds of mRNA codons

**Codons**	**Amino acid**	**Codons**	**Amino acid**
GCU, GCC, GCA, GCG	Alanine	CUU, CUC, CUA, CUG, UUA, UUG	Leucine
CGU, CGC, CGA, CGG, AGA, AGG	Arginine	AAA, AAG	Lysine
GAU, GAC	Aspartic acid	AUG	Methionine
AAU, AAC	Asparagine	UUU, UUC	Phenylalanine
UGU, UGC	Cysteine	CCU, CCC, CCA, CCG	Proline
GAA, GAG	Glutamic acid	UCU, UCC, UCA, UCG, AGU, AGC	Serine
CAA, CAG	Glutamine	ACU, ACC, ACA, ACG	Threonine
GGU, GGC, GGA, GGG	Glycine	UGG	Tryptophan
CAU, CAC	Histidine	UAU, UAC	Tyrosine
AUU, AUC, AUA	Isoleucine	GUU, GUC, GUA, GUG	Valine
UAA, UAG, UGA			

For the 64 codons illustrated in Table 1, their corresponding triplets of DNA are illustrated in Table 2.

**Table 2:** The corresponding triplets of 64 codons

**Codons**	**Corresponding triplets**	**Codons**	**Corresponding triplets**
GCU, GCC, GCA, GCG	GCT, GCC, GCA, GCG	CUU, CUC, CUA, CUG, UUA, UUG	CTT, CTC, CTA, CTG, TTA, TTG
CGU, CGC, CGA, 0020CGG, AGA, AGG	CGT, CGC, CGA, CGG, AGA, AGG	AAA, AAG	AAA, AAG
GAU, GAC	GAT, GAC	AUG	ATG
AAU, AAC	AAT, AAC	UUU, UUC	TTT, TTC
UGU, UGC	TGT, TGC	CCU, CCC, CCA, CCG	CCT, CCC, CCA, CCG
GAA, GAG	GAA, GAG	UCU, UCC, UCA, UCG, AGU, AGC	TCT, TCC, TCA, TCG, AGT, AGC
CAA, CAG	CAA, CAG	ACU, ACC, ACA, ACG	ACT, ACC, ACA, ACG
GGU, GGC, GGA, GGG	GGT, GGC, GGA, GGG	UGG	TGG
CAU, CAC	CAT, CAC	UAU, UAC	TAT, TAC
AUU, AUC, AUA	ATT, ATC, ATA	GUU, GUC, GUA, GUG	GTT, GTC, GTA, GTG
UAA, UAG, UGA	TAA, TAG, TGA		

Based on the above 64 triplets of DNA illustrated in Table 2, we define a new mapping *Ψ* to map each of these triplets into a different weight. Obviously, the mapping *Ψ* shall satisfy the following rule: for any two pairs of triplets (*X*_1_, *Y*_1_) and (*X*_2_, *Y*_2_), where *X*_1_, *Y*_1_, *X*_2_, and *Y*_2_ are all triplets, if the corresponding codons of *X*_1_ and *Y*_1_ code the same amino acid but the corresponding codons of *X*_2_ and *Y*_2_ code two different amino acids, then there shall be |*Ψ* (*X*_1_) − *Ψ* (*Y*_1_)| < |*Ψ* (*X*_2_) − *Ψ* (*Y*_2_)|. So, according to the above rule and for the sake of convenience, weights consist of amino acid and codon. Amino acid is the integer part of weight, and codon is the fractional part of weight. Alanine is defined as 1, arginine is defined as 2, and the rest can be done in the same manner. Codons of every amino acid are reordered, so the first codon of alanine's (GCT) weight value is 1.1. We design the detailed mapping rules of *Ψ* as illustrated in Table 3.

**Table 3:** **The mapping rules of *Ψ***

**Triplet**	**Corresponding weight**	**Triplet**	**Corresponding weight**
GCT	1.1	CTT	11.1
GCC	1.2	CTC	11.2
GCA	1.3	CTA	11.3
GCG	1.4	CTG	11.4
		TTA	11.5
		TTG	11.6
CGT	2.1	AAA	12.3
CGC	2.2	AAG	12.4
CGA	2.3		
CGG	2.4		
AGA	2.5		
AGG	2.6		
GAT	3.3	TTT	13.1
GAC	3.4	TTC	13.2
AAT	4.1	CCT	14.1
AAC	4.2	CCC	14.2
		CCA	14.3
		CCG	14.4
TGT	5.1	TCT	15.1
TGC	5.2	TCC	15.2
		TCA	15.3
		TCG	15.4
		AGT	15.5
		AGC	15.6
GAA	6.1	ACT	16.3
GAG	6.2	ACC	16.4
		ACA	16.5
		ACG	16.6
CAA	7.1	TGG	17.3
CAG	7.2		
GGT	8.1	TAT	18.1
GGC	8.2	TAC	18.2
GGA	8.3		
GGG	8.4		
CAT	9.1	GTT	19.1
CAC	9.2	GTC	19.2
		GTA	19.3
		GTG	19.4
ATT	10.1	ATG	20.1
ATC	10.2		
ATA	10.3		
TAA	21.1		
TAG	21.2		
TGA	21.3		

For example, from Table 3, we will have *Ψ* (GCT) = 1.1, *Ψ* (GCC) = 1.2, *Ψ* (ATG) = 20.1, etc., and in addition, we can propose a novel 2D graphical representation of DNA sequences as follows:

Let *G* = *g*_1_, *g*_2_, *g*_3_…*g*_*N*_ be an arbitrary DNA primary sequence, where *g*_*i*_ ∈ {A, T, G, C} for any *i* ∈ {1, 2,…, *N*}, and then, we can transform *G* into a sequence of triplets such as *G* = *t*_1_, *t*_2_, *t*_3_…*t*_*M*_, where *M* = [*N*/3] and *t*_*i*_ is a triplet of DNA for any *i* ∈ {1, 2,…, *M*}. Thereafter, we can define a new mapping Θ to map *G* into a plot set as illustrated in the formula (1).

(1) $$\Theta \left(G\right)=\left\{\left(1,\Psi \left(t_{1}\right)\right),\left(2,\Psi \left(t_{2}\right)\right),\ldots ,\left(M,\Psi \left(t_{M}\right)\right)\right\}$$

**Figure 1:**
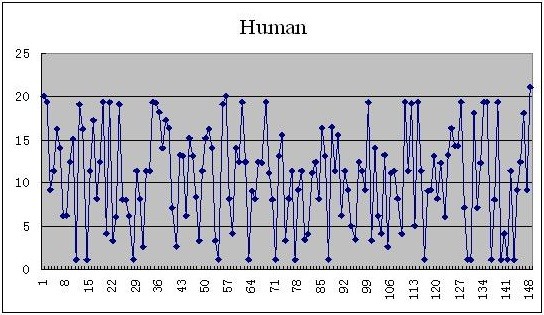
The 2D graphical representations of the complete coding sequences of β-globin genes of human.

**Figure 2:**
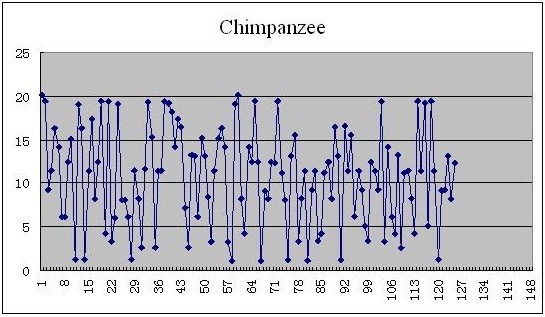
The 2D graphical representations of the complete coding sequences of β-globin genes of chimpanzee.

**Figure 3:**
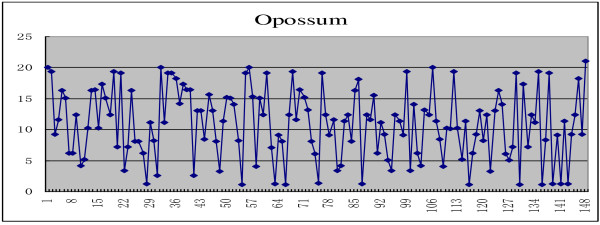
The 2D graphical representations of the complete coding sequences of β-globin genes of opossum.

As for the complete coding sequences of β-globin genes of 11 species illustrated in the Table 4, each of them can be mapped into a plot set by using the new given mapping Θ, and the 2D graphical representations corresponding to the complete coding sequences of β-globin genes of human, chimpanzee, and opossum are shown in Figures 1, 2, and 3, respectively.

**Table 4:** The complete coding sequences of β-globin genes of 11 species

**Species**	**Complete coding sequence**
Human	ATGGTGCACCTGACTCCTGAGGAGAAGTCTGCCGTTACTGCCCTGTGGGGCAAGGTGAACGTGGATGAAGTTGGTGGTGAGGCCCTGGGCAGGCTGCTGGTGGTCTACCCTTGGACCCAGAGGTTCTTTGAGTCCTTTGGGGATCTGTCCACTCCTGATGCTGTTATGGGCAACCCTAAGGTGAAGGCTCATGGCAAGAAAGTGCTCGGTGCCTTTAGTGATGGCCTGGCTCACCTGGACAACCTCAAGGGCACCTTTGCCACACTGAGTGAGCTGCACTGTGACAAGCTGCACGTGGATCCTGAGAACTTCAGGCTCCTGGGCAACGTGCTGGTCTGTGTGCTGGCCCATCACTTTGGCAAAGAATTCACCCCACCAGTGCAGGCTGCCTATCAGAAAGTGGTGGCTGGTGTGGCTAATGCCCTGGCCCACAAGTATCACTAA
Chimpanzee	ATGGTGCACCTGACTCCTGAGGAGAAGTCTGCCGTTACTGCCCTGTGGGGCAAGGTGAACGTGGATGAAGTTGGTGGTGAGGCCCTGGGCAGGTTGGTATCAAGGCTGCTGGTGGTCTACCCTTGGACCCAGAGGTTCTTTGAGTCCTTTGGGGATCTGTCCACTCCTGATGCTGTTATGGGCAACCCTAAGGTGAAGGCTCATGGCAAGAAAGTGCTCGGTGCCTTTAGTGATGGCCTGGCTCACCTGGACAACCTCAAGGGCACCTTTGCCACACTGAGTGAGCTGCACTGTGACAAGCTGCACGTGGATCCTGAGAACTTCAGGCTCCTGGGCAACGTGCTGGTCTGTGTGCTGGCCCATCACTTTGGCAAAG
Gorilla	ATGGTGCACCTGACTCCTGAGGAGAAGTCTGCCGTTACTGCCCTGTGGGGCAAGGTGAACGTGGATGAAGTTGGTGGTGAGGCCCTGGGCAGGCTGCTGGTGGTCTACCCTTGGACCCAGAGGTTCTTTGAGTCCTTTGGGGATCTGTCCACTCCTGATGCTGTTATGGGCAACCCTAAGGTGAAGGCTCATGGCAAGAAAGTGCTCGGTGCCTTTAGTGATGGCCTGGCTCACCTGGACAACCTCAAGGGCACCTTTGCCACACTGAGTGAGCTGCACTGTGACAAGCTGCACGTGGATCCTGAGAACTTCAAGCTCCTGGGCAATGTGCTGGTCTGTGTGCTGGCCCATCACTTTGGCAAAG
Black lemur	ATGACTTTGCTGAGTGCTGAGGAGAATGCTCATGTCACCTCTCTGTGGGGCAAGGTGGATGTAGAGAAAGTTGGTGGCGAGGCCTTGGGCAGGCTGCTGGTCGTCTACCCATGGACCCAGAGGTTCTTCGAGTCCTTTGGGGACCTGTCCTCTCCTTCTGCTGTTATGGGGAACCCTAAGGTGAAGGCCCATGGCAAGAAGGTGCTGAGTGCCTTTAGTGAAGGTCTGCATCACCTGGACAACCTCAAGGGCACCTTTGCTCAACTGAGTGAGCTGCACTGTGACAAGTTGCACGTGGATCCTCAGAACTTCACTCTCCTGGGCAACGTGCTGGTGGTTGTGCTGGCTGAACACTTTGGCAATGCATTCAGCCCGGCGGTGCAGGCTGCCTTTCAGAAGGTGGTGGCTGGTGTGGCCAATGCTCTGGCTCACAAGTACCACTGA
Norway rat	ATGGTGCACCTAACTGATGCTGAGAAGGCTACTGTTAGTGGCCTGTGGGGAAAGGTGAATGCTGATAATGTTGGCGCTGAGGCCCTGGGCAGGCTGCTGGTTGTCTACCCTTGGACCCAGAGGTACTTTTCTAAATTTGGGGACCTGTCCTCTGCCTCTGCTATCATGGGTAACCCCCAGGTGAAGGCCCATGGCAAGAAGGTGATAAATGCCTTCAATGATGGCCTGAAACACTTGGACAACCTCAAGGGCACCTTTGCTCATCTGAGTGAACTCCACTGTGACAAGCTGCATGTGGATCCTGAGAACTTCAGGCTCCTGGGCAATATGATTGTGATTGTGTTGGGCCACCACCTGGGCAAGGAATTCACCCCCTGTGCACAGGCTGCCTTCCAGAAGGTGGTGGCTGGAGTGGCCAGTGCCCTGGCTCACAAGTACCACTAA
House mouse	ATGGTGCACCTGACTGATGCTGAGAAGTCTGCTGTCTCTTGCCTGTGGGCAAAGGTGAACCCCGATGAAGTTGGTGGTGAGGCCCTGGGCAGGCTGCTGGTTGTCTACCCTTGGACCCAGCGGTACTTTGATAGCTTTGGAGACCTATCCTCTGCCTCTGCTATCATGGGTAATCCCAAGGTGAAGGCCCATGGCAAAAAGGTGATAACTGCCTTTAACGAGGGCCTGAAAAACCTGGACAACCTCAAGGGCACCTTTGCCAGCCTCAGTGAGCTCCACTGTGACAAGCTGCATGTGGATCCTGAGAACTTCAGGCTCCTAGGCAATGCGATCGTGATTGTGCTGGGCCACCACCTGGGCAAGGATTTCACCCCTGCTGCACAGGCTGCCTTCCAGAAGGTGGTGGCTGGAGTGGCCACTGCCCTGGCTCACAAGTACCACTAA
Goat	ATGCTGACTGCTGAGGAGAAGGCTGCCGTCACCGGCTTCTGGGGCAAGGTGAAAGTGGATGAAGTTGGTGCTGAGGCCCTGGGCAGGCTGCTGGTTGTCTACCCCTGGACTCAGAGGTTCTTTGAGCACTTTGGGGACTTGTCCTCTGCTGATGCTGTTATGAACAATGCTAAGGTGAAGGCCCATGGCAAGAAGGTGCTAGACTCCTTTAGTAACGGCATGAAGCATCTTGACGACCTCAAGGGCACCTTTGCTCAGCTGAGTGAGCTGCACTGTGATAAGCTGCACGTGGATCCTGAGAACTTCAAGCTCCTGGGCAACGTGCTGGTGGTTGTGCTGGCTCGCCACCATGGCAGTGAATTCACCCCGCTGCTGCAGGCTGAGTTTCAGAAGGTGGTGGCTGGTGTTGCCAATGCCCTGGCCCACAGATATCACTAA
Bovine	ATGCTGACTGCTGAGGAGAAGGCTGCCGTCACCGCCTTTTGGGGCAAGGTGAAAGTGGATGAAGTTGGTGGTGAGGCCCTGGGCAGGCTGCTGGTTGTCTACCCCTGGACTCAGAGGTTCTTTGAGTCCTTTGGGGACTTGTCCACTGCTGATGCTGTTATGAACAACCCTAAGGTGAAGGCCCATGGCAAGAAGGTGCTAGATTCCTTTAGTAATGGCATGAAGCATCTCGATGACCTCAAGGGCACCTTTGCTGCGCTGAGTGAGCTGCACTGTGATAAGCTGCATGTGGATCCTGAGAACTTCAAGCTCCTGGGCAACGTGCTAGTGGTTGTGCTGGCTCGCAATTTTGGCAAGGAATTCACCCCGGTGCTGCAGGCTGACTTTCAGAAGGTGGTGGCTGGTGTGGCCAATGCCCTGGCCCACAGATATCATTAA
Rabbit	ATGGTGCATCTGTCCAGTGAGGAGAAGTCTGCGGTCACTGCCCTGTGGGGCAAGGTGAATGTGGAAGAAGTTGGTGGTGAGGCCCTGGGCAGGCTGCTGGTTGTCTACCCATGGACCCAGAGGTTCTTCGAGTCCTTTGGGGACCTGTCCTCTGCAAATGCTGTTATGAACAATCCTAAGGTGAAGGCTCATGGCAAGAAGGTGCTGGCTGCCTTCAGTGAGGGTCTGAGTCACCTGGACAACCTCAAAGGCACCTTTGCTAAGCTGAGTGAACTGCACTGTGACAAGCTGCACGTGGATCCTGAGAACTTCAGGCTCCTGGGCAACGTGCTGGTTATTGTGCTGTCTCATCATTTTGGCAAAGAATTCACTCCTCAGGTGCAGGCTGCCTATCAGAAGGTGGTGGCTGGTGTGGCCAATGCCCTGGCTCACAAATACCACTGA
Opossum	ATGGTGCACTTGACTTCTGAGGAGAAGAACTGCATCACTACCATCTGGTCTAAGGTGCAGGTTGACCAGACTGGTGGTGAGGCCCTTGGCAGGATGCTCGTTGTCTACCCCTGGACCACCAGGTTTTTTGGGAGCTTTGGTGATCTGTCCTCTCCTGGCGCTGTCATGTCAAATTCTAAGGTTCAAGCCCATGGTGCTAAGGTGTTGACCTCCTTCGGTGAAGCAGTCAAGCATTTGGACAACCTGAAGGGTACTTATGCCAAGTTGAGTGAGCTCCACTGTGACAAGCTGCATGTGGACCCTGAGAACTTCAAGATGCTGGGGAATATCATTGTGATCTGCCTGGCTGAGCACTTTGGCAAGGATTTTACTCCTGAATGTCAGGTTGCTTGGCAGAAGCTCGTGGCTGGAGTTGCCCATGCCCTGGCCCACAAGTACCACTAA
*Gallus*	ATGGTGCACTGGACTGCTGAGGAGAAGCAGCTCATCACCGGCCTCTGGGGCAAGGTCAATGTGGCCGAATGTGGGGCCGAAGCCCTGGCCAGGCTGCTGATCGTCTACCCCTGGACCCAGAGGTTCTTTGCGTCCTTTGGGAACCTCTCCAGCCCCACTGCCATCCTTGGCAACCCCATGGTCCGCGCCCACGGCAAGAAAGTGCTCACCTCCTTTGGGGATGCTGTGAAGAACCTGGACAACATCAAGAACACCTTCTCCCAACTGTCCGAACTGCATTGTGACAAGCTGCATGTGGACCCCGAGAACTTCAGGCTCCTGGGTGACATCCTCATCATTGTCCTGGCCGCCCACTTCAGCAAGGACTTCACTCCTGAATGCCAGGCTGCCTGGCAGAAGCTGGTCCGCGTGGTGGCCCATGCCCTGGCTCGCAAGTACCACTAA

## 3. Similarity analysis of DNA sequence

Let *G* = *g*_1_, *g*_2_, *g*_3_…*g*_*N*_ be an arbitrary complete coding sequence, where *g*_*i*_ ∈ {A, T, G, C} for any *i* ∈ {1, 2,…, *N*}, and *G* = *t*_1_, *t*_2_, *t*_3_…*t*_*M*_ be its corresponding sequence of triplets, where *M* = [*N*/3] and *t*_*i*_ is a triplet of DNA for any *i* ∈ {1, 2,…, *M*}. Then, we define a function *δ* and let *δ* (*t*_*i*_) represent the total number of times that the triplet *t*_*i*_ repeats in the sequence of triplets *G* = *t*_1_, *t*_2_, *t*_3_…*t*_*M*_ for any *i* ∈ {1, 2,…, *M*}.

Let *T*_1_ = GCT, *T*_2_ = GCC, *T*_3_ = GCA, *T*_4_ = GCG, *T*_5_ = CGT, *T*_6_ = CGC, *T*_7_ = CGA, *T*_8_ = CGG, *T*_9_ = AGA, *T*_10_ = AGG, *T*_11_ = GAT, *T*_12_ = GAC, *T*_13_ = AAT, *T*_14_ = AAC, *T*_15_ = TGT, *T*_16_ = TGC, *T*_17_ = GAA, *T*_18_ = GAG, *T*_19_ = CAA, *T*_20_ = CAG, *T*_21_ = GGT, *T*_22_ = GGC, *T*_23_ = GGA, *T*_24_ = GGG, *T*_25_ = CAT, *T*_26_ = CAC, *T*_27_ = ATT, *T*_28_ = ATC, *T*_29_ = ATA, *T*_30_ = CTT *T*_31_ = CTC, *T*_32_ = CTA, *T*_33_ = CTG, *T*_34_ = TTA, *T*_35_ = TTG, *T*_36_ = AAA, *T*_37_ = AAG, *T*_38_ = TTT, *T*_39_ = TTC, *T*_40_ = CCT, *T*_41_ = CCC, *T*_42_ = CCA, *T*_43_ = CCG, *T*_44_ = TCT, *T*_45_ = TCC, *T*_46_ = TCA, *T*_47_ = TCG, *T*_48_ = AGT, *T*_49_ = AGC, *T*_50_ = ACT, *T*_51_ = ACC, *T*_52_ = ACA, *T*_53_ = ACG, *T*_54_ = TGG, *T*_55_ = TAT, *T*_56_ = TAC, *T*_57_ = GTT, *T*_58_ = GTC, *T*_59_ = GTA, *T*_60_ = GTG, *T*_61_ = ATG, *T*_62_ = TAA, *T*_63_ = TAG, and *T*_64_ = TGA.

Thereafter, according to Table 2, since there are a total of 64 triplets of DNA, then we can construct a set of 64 vectors {<*T*_1_, *δ* (*T*_1_)>, <*T*_2_, *δ* (*T*_2_)>,…, <*T*_64_, *δ* (*T*_64_)>} for the given sequence of triplets *G* = *t*_1_, *t*_2_, *t*_3_…*t*_*M*_ as follows: if *T*_*i*_ = *t*_*j*_ ∈ {*t*_1_, *t*_2_, *t*_3_,…*t*_*M*_}, then *δ* (*T*_*i*_) = *δ* (*t*_*j*_), else *δ* (*T*_*i*_) =0, for any *i* ∈ {1, 2,…, 64} and *j* ∈ {1, 2,…, *M*}.

For convenience, we call {<*T*_1_, *δ* (*T*_1_)>, <*T*_2_, *δ* (*T*_2_)>,…, <*T*_64_, *δ* (*T*_64_)>} as the triplet-repeat model set of *G*.

For any two given complete coding sequences *A* and *B*, suppose that their triplet-repeat model sets are {<*T*_1_, *X*_1_>, <*T*_2_, *X*_2_>,…, <*T*_64_, *X*_64_>} and {<*T*_1_, *Y*_1_>, <*T*_2_, *Y*_2_>,…, <*T*_64_, *Y*_64_>}, respectively. Then, on the basis of the 2D graphical representation given in the previous Section 2, we can define the weight deviation between the two DNA sequences *A* and *B* as the following formula (2) to measure the similarity between *A* and *B*.

(2) $$\mathrm{WD}\left(A,B\right)=\frac{\sum_{i=1}^{64}\left|X_{i}-Y_{i}\right|*\Psi \left(T_{i}\right)}{64}$$

**Figure 4:**
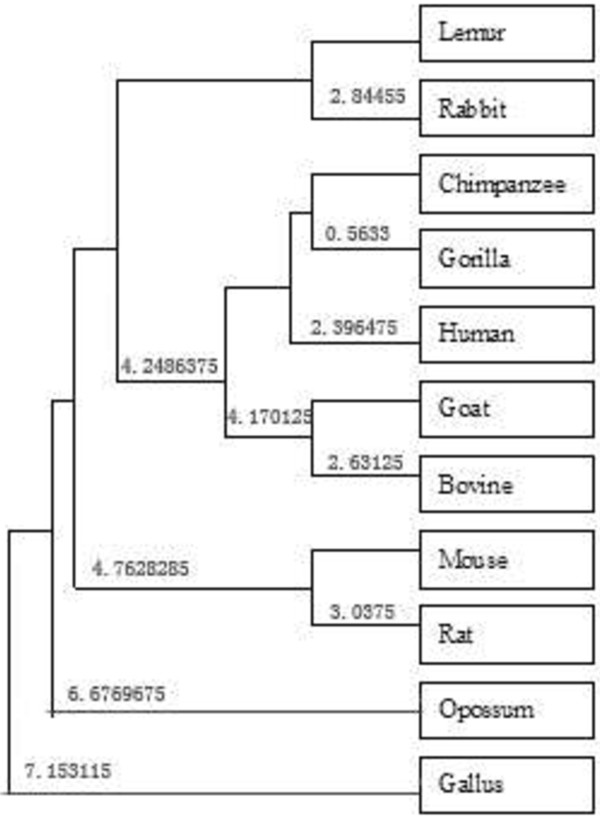
**Phylogenetic tree based on the similarity matrix (Table**5**).**

Obviously, the above formula (2) satisfies the fact that the smaller the weight deviation between the two DNA sequences *A* and *B*, the higher the degree of similarity of *A* and *B*. According to formula (2), the detailed similarity/dissimilarity matrix obtained for the coding sequences listed in Table 4 is illustrated in Table 5. Basing on the similarity matrix (Table 5) constructs a phylogenetic tree, which is shown in Figure 4.

**Table 5:** **The similarity/dissimilarity matrix for the coding sequences of Table**1**based on the weight deviation**

	**Human**	**Chimpanzee**	**Gorilla**	**Lemur**	**Rat**	**Mouse**	**Goat**	**Bovine**	**Rabbit**	**Opossum**	***Gallus***
Human	0	5.2500	4.3359	8.5891	10.670	9.7047	8.2219	8.1438	7.8281	15.6078	16.7109
Chimpanzee		0	1.1266	8.0297	10.645	9.6016	8.4375	9.3219	9.6000	14.2578	15.8734
Gorilla			0	7.8688	9.9625	8.6063	7.6734	8.5578	8.5547	13.9719	14.8781
Lemur				0	8.7219	9.5500	7.1328	9.3891	5.6891	12.9281	15.2000
Rat					0	6.0750	7.0484	9.3641	9.6578	13.5906	14.1219
Mouse						0	9.4953	9.2641	10.7984	12.3406	12.3688
Goat							0	5.2625	8.7219	11.9703	14.5359
Bovine								0	9.2906	12.5922	15.0234
Rabbit									0	14.8984	15.6953
Opossum										0	14.2750
*Gallus*											0

Observing Table 5, it is easy to find out that human, gorilla, and chimpanzee are most similar to each other, and the pairs like gorilla-chimpanzee (with weight deviation of 1.1266), human-gorilla (with weight deviation of 4.3359), and human-chimpanzee (with weight deviation of 5.2500) are the most similar species pairs, but *Gallus* and opossum are the most dissimilar to the others (with weight deviation bigger than 11). It is consistent with the fact that *Gallus* is not a mammal, whereas the others are mammals, and opossum is the most remote species from the remaining mammals. Similar results have been obtained in other papers by different approaches [2,5,7,9,33].

For testing the validity of our method, the existing results of the examination of the degree of similarity/dissimilarity of the coding sequences of β-globin genes of several species with the coding sequence of the human β-globin gene by means of approaches using alternative DNA sequence descriptors [2,5,7,9] are listed in Table 6 for comparison.

**Table 6:** The similarity/dissimilarity of the coding sequences

**Species**	**A**	**B**	**C**	**D**	**E**
Chimpanzee	5.2500	0.0144	14.00	0.005069	0.863
Gorilla	4.3359	0.0125	13.63	0.006611	0.339
Lemur	8.5891	-	31.75	0.030894	1.188
Rat	10.670	0.1377	41.65	0.015539	1.966
Mouse	9.7047	0.1427	30.27	0.015700	0.735
Goat	8.2219	0.1161	31.39	0.020980	0.311
Bovine	8.1438	0.0773	30.68	0.017700	2.489
Rabbit	7.8281	0.1332	35.575	0.015788	1.372
Opossum	15.6078	-	48.701	0.033363	6.322
*Gallus*	16.7109	-	70.46	0.025801	7.170

From Table 6, we can find that the pairs like human-gorilla and human-chimpanzee are the two most similar species pairs when adopting (A) the method of our work, (B) the method of [2], (C) the method of [5], and (D) the method of [7], which is in accordance with the fact that gorilla and chimpanzee are the two most closest species of human, but when adopting (E) the method of [9], the most similar species pair is human-goat, which is obviously not correct. In addition, the pairs like human-*Gallus* and human-opossum are the two most dissimilar species pairs when adopting (A) the method of our work, (C) the method of [5], and (E) the method of [9], which is in accordance with the fact that *Gallus* is not a mammal, whereas the others are mammals, and opossum is the most remote species from the remaining mammals. However, when adopting (D) the method of [7], the two most dissimilar species pairs are human-opossum and human-lemur, which is obviously not reasonable also.

## 4. Conclusion

In this paper, we propose a new 2D graphical representation for DNA sequences based on triplets, and associating with a newly introduced concept of weight of triplets and a newly designed measure of similarity named weight deviation, we propose a new method to make similarity analysis of DNA sequences, in which no matrix computation is needed and reasonable and useful approaches for both computational scientists and molecular biologists to effectively analyze DNA sequences can be provided at the same time.

## Competing interests

The authors declare that they have no competing interests.
